# Hearing Status in Patients with Type 2 Diabetes Mellitus According to Blood-Sugar Control: A Comparative Study

**Published:** 2018-07

**Authors:** Shadman Nemati, Rasool Hassanzadeh, Mojtaba Mehrdad, Sahar Sajedi Kia

**Affiliations:** 1 *Rhino-sinus, Ear and Skull base Diseases Research Center, Guilan University of Medical Sciences, Rasht, Iran . *; 2 *Department of Endocrinology, Razi Hospital, Guilan University of Medical Sciences, Rasht, Iran.*; 3 *Department of Otolaryngology, Head and Neck Surgery and Research Center, Amiralmomenin Hospital, Guilan University of Medical Sciences, Rasht, Iran.*

**Keywords:** Diabetes mellitus type 2, Glycated Hemoglobin a, Hearing loss, Otoacoustic emissions, Sensorineural, Tinnitus, Vertigo

## Abstract

**Introduction:**

It seems that diabetes mellitus (DM) can affect the auditory system due to neuropathy, micro-vascular complications, and hearing cell damage during hyperglycemic states. In the current study, we aimed to compare hearing status in patients with type 2 DM (T2DM) according to their blood-sugar control status.

**Materials and Methods:**

This cross-sectional study was carried out in 104 patients with T2DM attending the diabetic clinics of Guilan University of Medical Sciences within a period of 1 year (2014–2015). One group consisted of 52 patients with poor control and the other consisted of patients with moderate-to-good control (according to glycated hemoglobin [HbA1c] level). All subjects underwent pure tone audiometry (PTA) and distortion product otoacoustic emission (DPOAEs) assessments. A hearing threshold higher than 20 dB and a signal-to-noise ratio ≤3 in each frequency were considered abnormal.

**Results:**

In PTA, poorly controlled patients showed more frequent hearing loss compared with the well-controlled group, especially at higher frequencies (8 kHz: 67.3% vs 46.2% [P=0.029]; 10 kHz: 46.2% vs 21.2% [P=0.025]). Also, patients in the poorly controlled group had worse cochlear function according to the DPOAE test (4 kHz: 32.7% vs 17.3% [P= 0.002] and 8 kHz: 70.6% vs 40.4% [P=0.006]).

**Conclusion:**

DM and poor control status of diabetes can affect hearing sensation and cause hearing loss, especially at high frequencies. According to our findings, it seems that diabetic patients with a duration of diabetes >10 years, diabetic complications, poor control status or comorbidities should undergo both endocrine and audiologic follow-up to prevent greater sensory neural hearing loss.

## Introduction

The number of people with diabetes mellitus (DM) is growing for a number of reasons including population growth, aging, urbanization, and the increasing prevalence of obesity and physical inactivity. Rational planning and allocation of resources rely on quantifying the prevalence of diabetes and the number of people affected by diabetes, now and in the future. Type 2 DM (T2DM) is a complex disease with both metabolic and vascular complications that affected about 285 million worldwide in 2010, and is predicted to rise to 438 million by the end of 2030 within the 20–70-year age group (1). In the Islamic Republic of Iran, despite the existence of a national diabetes prevention and control program, we still face a large burden of DM (2). According to recent records, the current prevalence of DM in Iran is 10.3% (3).

The influence of glycosides and lipids and metabolic complications on vestibular and auditory systems have been considered the main etiologic factors related to hearing impairment, tinnitus, and dizziness in DM (4). Therefore, the diabetic population are in a high-risk group for auditory complications (5). Jordao first described the relationship between DM and hearing loss (6), and this association is still controversial. Despite the fact that most studies reported bilateral progressive high-frequency sensorineural hearing loss (SNHL) in diabetic patients (7-9), others revealed that hearing was not affected in patients with diabetes (10-12). Glycosylated hemoglobin (HbA1C) is one of the indicators for glycemic control status in patients with DM. However, there is no systematic association between its elevated levels and increased hearing thresholds. Thus, direct evidence that poor metabolic control status in diabetes results in a greater degree of SNHL remains to be proven (13). Although some studies have revealed the occurrence of SNHL in DM, there is still disagreement among some authors about this relationship (14-15).Hearing loss and T2DM are significant health problems, so it is worthwhile to study the relationship between the two (16). The purpose of the current study was to examine whether, among diabetic patients, the degree of hearing loss is associated with glycemic control (HbA1C levels) and other factors related to control of diabetes. The second objective was to correlate the audiological findings with factors such as duration of DM, gender, comorbidities, and the presence of other diabetes complications.

## Materials and Methods

This cross-sectional study included 104 patients with T2DM (52 poorly controlled patients and 52 cases with moderate-to-good control) who were selected from adult diabetic patients attending the diabetic clinics of our university hospitals during 2014–2015. Patients were selected using a systematic random sampling technique (every other patient who met the inclusion criteria was chosen) and referred to the Ear, Nose, and Throat (ENT) clinic of our hospital for a hearing assessment. All investigations were performed in accordance with the Declaration of Helsinki on biomedical studies involving human subjects, and informed consent was obtained from all participants. This study was approved by the Clinical Research Ethics Committee of Guilan University of Medical Sciences (GUMS).

Well-controlled patients were defined as those who had met the following criteria: fasting blood sugar (FBS) ˂130 mg/dl, 2-hour postprandial blood sugar (2hrPPBS) ˂180 mg/dl, and HbA1c ˂7%. Patients aged 20–60 years diagnosed with T2DM according to World Health Organization criteria and with no previous history of ear disease were selected for the study. To rule out the influence of age‑related changes in hearing status, this study was performed in DM subjects aged ˂60 years, and the groups were matched in terms of age. Exclusion criteria were history of chronic ear disease such as chronic suppurative otitis media, history of ototoxic drug intake (such as gentamycin, quinidine, high dose aspirin and furosemide) in the past 3 months, abnormal otoscopic and tympanometric examinations, heavy smokers (more than one pack per day), occupational noise exposure, severe or uncontrolled DM, heavy alcohol consumption, and neurologic disorders such as multiple sclerosis.

*Examination procedure*

A complete detailed medical history (retinopathy, nephropathy, neuropathy, angiopathy, diabetic ketoacidosis, and diabetic hyperosmolar coma) was taken. All subjects underwent biochemical investigations such as postprandial blood sugar (PPBS), FBS, HbA1c, total cholesterol, triglyceride, and low-density lipoprotein (LDL). A clinical and ENT examination was carried out and a history of otologic disease such as tinnitus and hearing loss was taken. An audiological examination including tympanometry and acoustic reflex was performed in all cases to rule out middle-ear disorders. Pure tone audiometry (PTA) was measured at 0.25, 0.5, 1, 2, 4, and 8 kHz and also, high-frequency PTA was measured at 10, 12, 14 and 16 kHz to detect the hearing threshold at each given frequency using an AC40 clinical audiometer (Madsen, Denmark) in a sound-isolated room, standardized according to the manufacturer’s instructions. Hearing loss was defined as the threshold of PTA at any frequency >20 dB HL. Speech reception threshold (SRT) and speech discrimination score (SDS) were investigated using one- and two-syllable words of equal stress (Spondees), and thresholds more than 25 dB HL and SDS lower than 75% were considered abnormal.

Distortion product otoacoustic emissions (DPOAEs) were obtained using the Madsen Capella DPOAE System. Adequacy of probe fit was inspected prior to the commencement of data acquisition. A series of simultaneous pure tone pairs, of frequencies f1 and f2, at intensities of 65 and 55 dB SPL, respectively, were delivered to the test ear. All primary tones were maintained within ±1 dB of the set intensity. These stimulus intensity levels were chosen based on recommendations concerning optimal results in humans. To achieve optimal DPOAE results, the test frequency ratio (f2/f1) was set at 1.21. For each test ear, a DP-gram that plots intensity of the 2f1-f2 distortion product amplitude (and mean noise floor) against f2 frequency was obtained. To record the DPOAEs, the default protocol recommended by the manufacturer was used. Intensity of the 2f1-f2 distortion product amplitude at frequencies of 0.75–8 kHz was recorded and a signal/ noise ratio (SNR) ≥3 dB was considered as normal result.

The main outcome variable was to profile the PTA and DPOAE findings in diabetic subjects. However, there were many variables of interest such as age, gender, glycemic control (HbA1C level), glycemic status (FBS and 2hrPPBS), duration of disease, tinnitus, vertigo and comorbid disease. After data collection, statistical analysis was performed using the Statistical Package for Social Sciences (SPSS) version 19. The Kolmogorov-Smirnov test was used to evaluate normalcy of distribution of the data. We used the Mann-Whitney test or the Chi-square Fisher Exact and Kruskal Wallis tests to compare the data in the groups. To control the effects of various parameters, such as age, and duration of disease for example, we used multivariable analysis. The level of significance was considered to be <0.05.

## Results

The study included 104 subjects with a mean age of 53.74±4.8 years (range, 39–60 years), among whom 76% were female. The participants were divided into two groups according to HbA1c level (poor control and good control). Age distribution and HbA1c level, FBS and 2hrPPBS according to diabetes's control status are summarized in Table 1.

**Table 1:** Age, Hb A1C^a^, FBS^b^, and 2hrPPBS^c^ levels in two groups of DM^d^ patients; well-controlled and poorly controlled groups

**Variables**	**Diabetes's control status**	**Mean**	**Standard deviation**	**Minimum**	**Maximum**	**Percentile**			**P-value**
						**25**	**50**	**75**	
Age (year)	Good control	54.25	5.85	10	65	50	54	60	0.867
	Poor control	54.06	5.79						
HbA1C (%)	Good control	7.03	0.80	4.7	12.5	7.20	8.10	9.50	0.0001*
	Poor control	9.93	1.03						
FBS (mg/dl)	Good control	147.04	26.09	54	346	123.25	144	177.25	0.09
	Poor control	165.89	73.29						
2hrPPBS (mg/dl)	Good control	197.25	62.54	90	500	169.50	228	285.75	0.0001*
	Poor control	269.79	92.95						

* : P<0.05 was considered statistically significant,

a : HbA1c: Glycosylated hemoglobin,

b : 2 hrPPBS: 2-hour postprandial blood sugar,

c : FBS: Fasting blood sugar,

d : DM: Diabetes mellitus

Using the Chi-square test, the number of males among well-controlled diabetic patients was 15.4% (8/52) compared with 32.7% in the poorly controlled group (17/52); which was statistically significant (P˂0.039).

For all frequencies, except 0.25 and 0.5 kHz, most of the patients demonstrated normal hearing status (89.4% in the right ear and 91.3% in the left ear); in addition, at 1- and 2-kHz frequencies, more than 75% of subjects had normal hearing. At higher frequencies, the rate of hearing loss increased. At frequencies of 4 and 8 kHz, this could still be investigated with a conventional PTA test, and 56–59.5% and 43–54.5% of subjects had normal hearing status, respectively. However, at frequencies of 9, 10, 11, and 12 kHz, which are measured by high-frequency PTA, approximately 65%, 70–75%, 85% and 98% of patients demonstrated hearing loss (Table.2).

**Table 2:** Frequency of normal hearing status in different frequencies

**Frequency (Hz)**	**Normal hearing status**	
	**Right ear, No. (%)**	**Left ear, No. (%)**
250	93 (89.4%)	95 (91.3%)
500	93 (89.4%)	95 (91.3%)
1,000	86 (82.7%)	92 (88.5%)
2,000	74 (71.2%)	78 (75%)
4,000	58 (55.8%)	62 (59.6%)
8,000	57 (54.8%)	45 (43.3%)
9,000	36 (34.6%)	37 (35.6%)
10,000	28 (26.9%)	33 (31.7%)
11,000	16 (15.4%)	17 (16.7%)
12,000	2 (1.9%)	2 (1.9%)

Chi-square and Fisher Exact Tests revealed statistically significant differences in hearing loss at the 9-kHz frequency between well and poorly controlled groups (P=0.002). However, at frequencies of 0.25 and 0.5 kHz in the left ear, two patients in the poorly controlled group and nine patients in the well-controlled group had hearing loss; however, due to the small sample size, this difference cannot be considered significant. According to level of HbA_1_c, in the poorly controlled group, hearing loss was more significant at the 8, 9, 10, and 11 kHz frequencies compared with the well-controlled group (P˂0.029) (Table.3).

**Table 3:** Hearing status in different frequencies according to PTA^a^ test in the study groups.

**Frequency (Hz)**	**Hearing status**	**Diabetes's control status**				**P-value**	
		**Good control**		**Poor control**			
		**Right ear, No. (%)**	**Left ear, No. (%)**	**Right ear, No. (%)**	**Left ear, No. (%)**	**Right ear **	**Left ear **
250	Normal	43 (82.7%)	46 (88.5%)	50 (96.2%)	49 (94.2%)	0.026	0.244
	Abnormal	9 (17.3%)	6 (11.5%)	2 (3.8%)	3 (5.8%)		
500	Normal	43 (82.7%)	46 (88.5%)	50 (96.2%)	49 (94.2%)	0.026	0.244
	Abnormal	9 (17.3%)	6 (11.5%)	2 (3.8%)	3 (5.8%)		
1,000	Normal	44 (84.6%)	47 (90.4%)	42 (80.8%)	45 (86.5%)	0.604	0.539
	Abnormal	8 (15.4%)	5 (9.6%)	10 (19.2%)	7 (13.5%)		
2,000	Normal	36 (69.2%)	40 (76.9%)	38 (73.1%)	38 (73.1%)	0.665	0.651
	Abnormal	16 (30.8%)	12 (23.1%)	14 (26.9%)	14 (26.9%)		
4,000	Normal	29 (55.8%)	29 (55.8%)	29 (55.8%)	33 (63.5%)	0.999	0.424
	Abnormal	23 (44.2%)	23 (44.2%)	23 (44.2%)	19 (35.5%)		
8,000	Normal	28 (53.8%)	28 (53.8%)	29 (55.8%)	17 (32.7%)	0.844	0.029
	Abnormal	24 (46.2%)	24 (46.2%)	23 (44.2%)	35 (67.3%)		
9,000	Normal	19 (36.5%)	22 (42.3%)	17 (32.7%)	15 (28.8%)	0.082	0.006
	Abnormal	11 (21.2%)	8 (15.4%)	21 (40.4%)	23 (44.2%)		
10,000	Normal	13 (25%)	19 (36.5%)	15 (28.2%)	14 (26.9%)	0.244	0.025
	Abnormal	17 (32.7%)	11 (21.2%)	23 (44.2%)	24 (46.2%)		
11,000	Normal	7 (13.5%)	12 (23.1%)	9 (17.3%)	5 (9.6%)	0.257	0.011
	Abnormal	23 (44.2%)	18 (34.6%)	29 (55.8%)	33 (63.5%)		
12,000	Normal	1 (1.9%)	0 (0%)	1 (1.9%)	2 (3.8%)	0.253	0.106
	Abnormal	29 (55.8%)	30 (57.7%)	37 (71.2%)	36 (69.2%)		

a Pure tone audiometric

In the DPOAE test, most patients (>64–74%) had normal test results at a frequency ˂6 kHz, but at the 8-kHz frequency, approximately 46% of patients were normal and more than 53% were abnormal. The Chi-square test was used and showed a statistically significant difference at frequencies of 0.75, 2, and 3 kHz 

in the left ear and at 1 kHz in right ear (P˂0.05), and the difference was most pronounced at the 8-kHz frequency in both ears (P˂0.006), indicating that patients in the poorly controlled group had worse cochlear function according to the DPOAE test (Table.4).

**Table 4:** Hearing status in different frequencies according to DPOAE^a^ test in the study groups

**Frequency (Hz)**	**Hearing status**	**Diabetes's control status**				**P-value**	
		**Good control**		**Poor control**			
		**Right ear,** **No. (%)**	**Left ear, No. (%)**	**Right ear, No. (%)**	**Left ear, No. (%)**	**Right ear**	**Left ear**
750	Normal	27 (51.9%)	34 (65.4%)	24 (47.1%)	21 (42.9%)	0.622	0.023*
	Abnormal	25 (48.1%)	18 (34.6%)	27 (52.9%)	28 (57.1%)		
1,000	Normal	41 (78.8%)	39 (75%)	30 57.7%)	33 (63.5%)	0.02*	0.202
	Abnormal	11 (21.2%)	13 (25%)	22 (42.3%)	19 (36.5%)		
1,500	Normal	43 (82.7%)	47 (90.4%)	39 (75%)	40 (76.9%)	0.337	0.063
	Abnormal	9 (17.3%)	5 (9.6%)	13 (25%)	12 (23.1%)		
2,000	Normal	43 (82.7%)	47 (90.4%)	37 (71.2%)	39 (75%)	0.163	0.038*
	Abnormal	9 (17.3%)	5 (9.6%)	15 (28.8%)	13 (25%)		
3,000	Normal	39 (78%)	42 (80.8%)	36 (69.2%)	32 (61.5%)	0.282	0.030*
	Abnormal	11 (22%)	10 (19.2%)	16 (30.8%)	20 (38.5%)		
4,000	Normal	41 (78.8%)	43 (82.7%)	38 (73.1%)	35 (67.3%)	0.491	0.07
	Abnormal	11 (21.2%)	9 (17.3%)	14 (26.9%)	17 (32.7%)		
6,000	Normal	36 (69.2%)	38 (73.1%)	31 (59.6%)	39 (75%)	0.306	0.823
	Abnormal	16 (30.8%)	14 (26.9%)	21 (40.4%)	13 (25%)		
8,000	Normal	33 (63.5%)	31 (59.6%)	15 (29.4%)	17 (32.7%)	0.001*	0.006*
	Abnormal	19 (36.5%)	21 (40.4%)	36 (70.6%)	35 (76.3%)		

a Distortion product otoacoustic emissions

To evaluate the association between hearing loss and existence of other complications related to diabetes (such as diabetic retinopathy, nephropathy, diabetic foot, for example), patients were divided into two groups: with or without complications. The results showed that at a frequency of 8 kHz in the left ear, 30 subjects among those with diabetic complications and 29 among patients without complications had hearing loss, while at a frequency of 11 kHz, 22 cases with diabetic complications and 29 patients without complications had hearing loss (P˂0.036).

In this study, 57 patients (54.8%) had comorbidities such as hypertension, thyroid dysfunction, and rheumatological diseases, for example; of these 24 (42.6%) were in the well-controlled group and 33 (63.5%) were in the poorly controlled group. No significant difference was observed (P˂0.076) (Table.5). 

**Table 5:** Frequency of diabetic comorbidities according to diabetes control status

**Comorbidities**	**Diabetes's control status**		**Total** **No. (%)**	**P-value** ^*^
	**Good control, No. (%)**	**Poor control, No. (%)**		
**+**	28 (53.8%)	19 (36.5%)	47 (45.2%)	0.076
**_**	24 (46.2%)	33 (63.5%)	57 (54.8%)	

* : P-value < 0.05 was considered statistically significant

To assess the association between the existence of comorbidities in diabetic patients and hearing loss, patients with and without comorbidities in two separate groups were investigated at different frequencies. The results showed that there was significant difference at frequencies of 10 and 11 kHz in the right ear and at the 8-kHz frequency in the left ear between these two groups (P=0.003, P=0.032, and P=0.008, respectively). Among all patients, only 20 (19.2%) had tinnitus; 13 of whom were in the poorly controlled group and seven of whom were in the well-controlled group. The Chi-square test was used to analyze the association between tinnitus and glycemic control status. No statistically significant difference was observed between these groups (P˂0.135). In addition, 27 subjects (26%) experienced episodes of true vertigo; 13 of whom were in the well-controlled group with the remaining number in the poorly controlled group, with no statistically significant differences.

To assess the effect of the duration of diabetes on hearing status, patients were divided into two groups: those with a known diabetes duration of ≥10 years and those who had been diagnosed for less than 10 years. At frequencies of 10 and 11 kHz, statistically significant differences were observed between hearing loss and duration of illness ≥10 years, in both ears (P=0.0001) (Table.6).

**Table 6:** Hearing status in different frequencies according to PTA^a^ test in subgroups of diabetic patients based on duration of diagnosis

**Frequency (Hz)**	**Hearing status**	**Duration of diabetes**				**P-value**	
		**<10 years**		**≥10 years**			
		**Right ear No. (%)**	**Left ear No. (%)**	**Right ear No. (%)**	**Left ear No. (%)**	**Right ear **	**Left ear **
1,000	Abnormal	17 (24.3%)	10 (14.3%)	1 (2.9%)	2 (5.9%)	0.07	0.177
	Normal	53 (75.7%)	60 (85.7%)	33 (97.1%)	32 (94.1%)		
2,000	Abnormal	22 (31.4%)	20 (28.6%)	8 (23.5%)	6 (17.6%)	0.404	0.224
	Normal	48 (68.6%)	50 (71.4)	26 (76.5%)	28 (82.4%)		
4,000	Abnormal	31 (44.3%)	28 (40%)	15 (44.1%)	14 (41.2%)	0.987	0.909
	Normal	39 (55.7%)	42 (60%)	19 (55.9%)	20 (58.8%)		
10,000	Abnormal	25 (35.7%)	22 (31.4%)	15 (44.1%)	13 (38.2%)	0.0001	0.0001
	Normal	11 (15.7%)	14 (20%)	17 (50%)	19 (55.9%)		
11,000	Abnormal	28 (40%)	27 (38.6%)	24 (70.6%)	24 (70.6%)	0.0001	0.0001
	Normal	8 (11.4%)	9 (12.9%)	8 (23.5%)	8 (23.5%)		

a Pure tone audiometric

In addition, in order to evaluate the effect of age on hearing status, the patients were divided into two groups: 25–45 years and 45–60 years of age. The results of the DPOAEs tests revealed that for all frequencies except 2 kHz, the greatest hearing loss was seen in the 45–60-year age group in the left ear, and the majority of patients (63.4%) demonstrated hearing loss at the 8-kHz frequency (P˂0.004). These results in the right ear were such that at the 2-, 4-, and 8-kHz frequencies, 25.4%, 28.2%, and 57.7% patients in 45–60-year age group, respectively, demonstrated hearing loss, but the results were not statistically significant.

**Fig 1:**
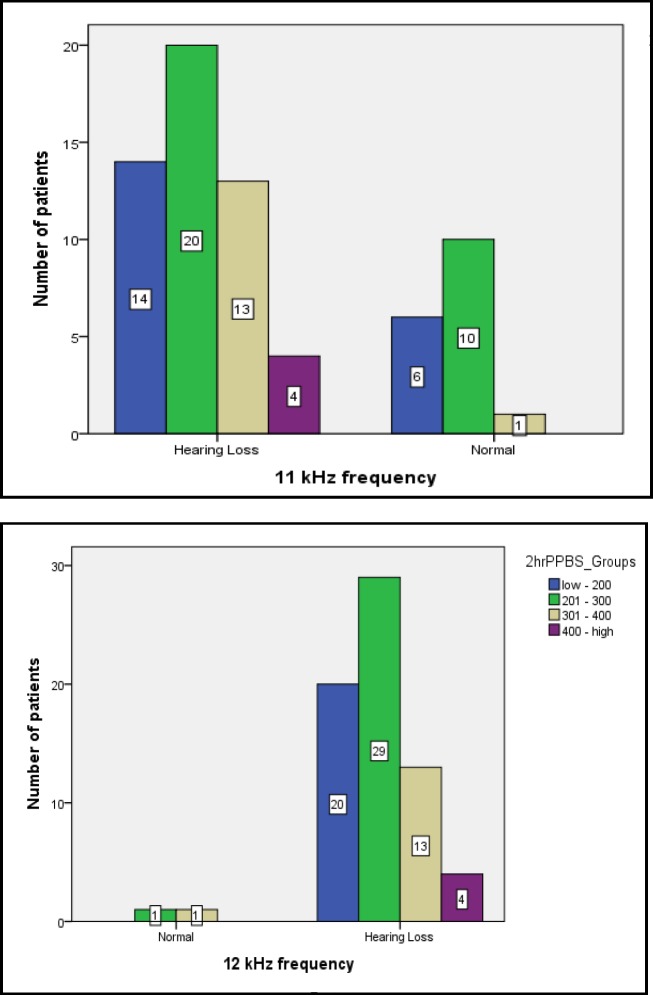
Association between 2hrPPBS levels and hearing loss at 11- and 12-kHz frequencies

**Fig 2:**
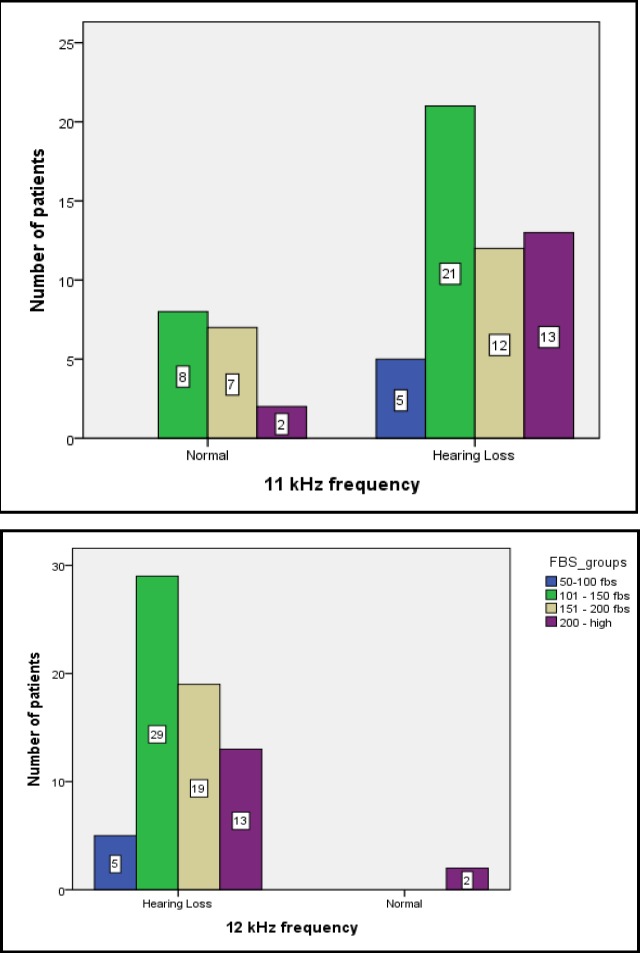
Association between FBS levels and hearing loss at 11- and 12-kHz frequencies.

To assess the association between FBS and 2hrPPBS levels and hearing loss, the patients were divided into different groups according to FBS and 2hrPPBS levels: FBS=50–100, 101–150, 151–200, and ˃200 mg/dl and 2hrPPBS ˂200, 200–300, 301–400, and ˃400 mg/dl. At high frequencies (>10 kHz), a statistically significant difference was seen between the different groups. The greatest associations were seen when FBS was 151–200 and ˃200 mg/dl, respectively, and also when 2hrPPBS was 301–400 and ˃400 mg/dl, respectively (Fig.1,2).

## Discussion

Jordao first described the association between DM and hearing loss (6), which is still controversial and attracts considerable attention. In a review of the literature, a number of studies are published that assessed the effect of DM on hearing acuity, but to the best of our knowledge, very few studies assessed the association between glycemic control status and hearing loss, or compared so many other variables in poorly controlled and well-controlled diabetic patients.

For all frequencies except 0.25 and 0.5 kHz, most patients demonstrated normal hearing status, while at frequencies of 1 and 2 kHz, more than 75% of subjects had normal hearing .As the frequency increased, the rate of hearing loss also increased, such that at frequencies of 4 and 8 kHz, this could still be investigated with the usual PTA test, whereby 56–59.5% and 43–54.5% of subjects had normal hearing status, respectively. However, at frequencies of 9, 10, 11, and 12 kHz, which can be measured using high-frequency PTA, approximately 65%, 70–75%, 85%, and 98% of patients had hearing loss, respectively. These results show that high-frequency PTA is more accurate than the usual PTA test for early screening, and it may be helpful for the diagnosis of subclinical cases. Some studies revealed that higher frequencies were affected more in diabetic patients (17-19), which is consistent with our study. On the other hand, other studies have reported that all the frequencies are similarly affected (7).

The results of the DPOAE test in the well-controlled and poorly controlled groups showed a statistically significant difference at frequencies of 0.75, 2, and 3 kHz in the left ear and at 1 kHz in the right ear; the difference was most pronounced at the 8-kHz frequency in both ears. This means that patients in the poorly controlled group had worse cochlear function according to the DPOAE test. Lisowska et al. found that the amplitudes of DPOAE were lower in T2DM patients in comparison with the control group. However Park recorded only 8-kHz decreases on DPOAE amplitudes in patients with diabetes compared with the control group (20,21). In the same study, the DPOAE amplitudes of patients with controlled and uncontrolled diabetes were evaluated, and significant differences were seen at 6 and 8 kHz, which is consistent with our study. No statistically significant difference was found in DPOAE measurements between diabetic patients with metabolically controlled or uncontrolled in the study by Bayindir et al. (22). Amplitudes of DPOAEs in our study were not evaluated, and we used the signal-to-noise ratio as the diagnostic criteria, as in the studies of Ferreira et al. and Joshi et al. (23,24).

Statistically significant differences were seen in hearing loss at a frequency of 9 kHz between the well-controlled and poorly controlled groups. However, at frequencies of 0.25 and 0.5 kHz in the left ear, two patients in the poorly controlled group and nine patients in the well-controlled group had hearing loss; however, due to low volume of samples the difference was not statistically different. It must also be added that this insignificant difference is not clinically meaningful. This was also confirmed by the level of HbA_1_c, such that in the poorly controlled group, at frequencies of 8, 9, 10, and 11 kHz, this hearing loss was more significant than that in the well-controlled group. Panchu et al. reported that the lack of glycemic control reveals a positive correlation with the extent of hearing loss at all frequencies when compared with the good-glycemic control patients (25).

No significant difference was observed between the presence tinnitus the status of diabetic control in our study. Consistent with our study, Gibrin et al. also reported no statistically significant differences between patients with DM and the control group in terms of tinnitus (26). Furthermore, we found no significant correlation between episodes of true vertigo and good control of diabetes. Some authors have stated secondary vestibulocochlear changes as a consequence of DM and hyperinsulinemia (20,27-28). The hair cells and the central vestibular system are sensitive to changes caused by DM, while experimental studies reveal that small variations in glucose and plasma insulin affect the labyrinth (29,30).

The results of DPOAE tests revealed that for all frequencies except 2 kHz, the greatest hearing loss was seen in the 50–65-year group in the left ear, and that most patients (63.4%) demonstrated statistically significant hearing loss at the 8-kHz frequency. However, a study by Panchu et al. showed that the effect of age on auditory thresholds of diabetic patients was statistically insignificant (25).

The present study revealed that at frequencies of both 10 and 11 kHz, highly statistically significant differences were seen between hearing loss and duration of illness ≥10 years, in both ears. Similar results have not been reported in other studies (25). Also, at high frequencies (>10 kHz), a statistically significant difference was seen between the different groups, and the greatest associations were seen when FBS was ˃150 and also when 2hrPPBS was ˃300 mg/dl. These findings are relatively similar to those reported by Panchu et al. in terms of FBS, but are not compatible in terms of 2hrPPBS (25). Joshi et al. reported that mean DPOAE amplitude and SNR were not affected by the duration of diabetes (24), and they were not significantly elevated with duration of diabetes.

Our study showed that at frequencies of 8 kHz and 11 kHz in the left ear, patients with diabetic complications had statistically significantly greater hearing loss compared with patients without complications. Naini et al. also reported 42% hearing loss at high frequencies in patients with diabetic complications (31), although the hearing level of the patients without complications was normal at all frequencies.

Our results show that there is a significant difference at 10 and 11 kHz frequencies in the right ear and at the 8-kHz frequency in the left ear between patients with and without comorbidities. Swaminathan et al. reported an increased association of hearing loss in people with high total cholesterol, high triglycerides, and high LDL levels (32), which is compatible with our study. These abnormalities in hearing status can be due to changes in the cochlea, including thickened vessels of the stria vascularis, atrophy, and loss of outer hair cells without loss of spiral ganglion cells, as described by Fukushima et al. (33).

## Conclusion

According to the findings of our study, it seems that diabetic patients with a duration of diabetes >10 years, patients with other diabetic complications, poorly controlled patients or patients with comorbidities should undergo audiologic and endocrine follow-up to prevent greater sensory neural hearing loss. We also propose more comprehensive and detailed studies with a greater sample size to further clarify these associations.
